# Skeletal Muscle Regeneration in Cardiotoxin-Induced Muscle Injury Models

**DOI:** 10.3390/ijms232113380

**Published:** 2022-11-02

**Authors:** Yanjie Wang, Jianqiang Lu, Yujian Liu

**Affiliations:** School of Kinesiology, Shanghai University of Sport, Shanghai 200438, China

**Keywords:** cardiotoxin injury, mice, skeletal muscle, regeneration, mechanism

## Abstract

Skeletal muscle injuries occur frequently in daily life and exercise. Understanding the mechanisms of regeneration is critical for accelerating the repair and regeneration of muscle. Therefore, this article reviews knowledge on the mechanisms of skeletal muscle regeneration after cardiotoxin-induced injury. The process of regeneration is similar in different mouse strains and is inhibited by aging, obesity, and diabetes. Exercise, microcurrent electrical neuromuscular stimulation, and mechanical loading improve regeneration. The mechanisms of regeneration are complex and strain-dependent, and changes in functional proteins involved in the processes of necrotic fiber debris clearance, M1 to M2 macrophage conversion, SC activation, myoblast proliferation, differentiation and fusion, and fibrosis and calcification influence the final outcome of the regenerative activity.

## 1. Introduction

Skeletal muscle, the main organ of systemic metabolism in the body, is composed of differentiated fibers and displays a strong ability to regenerate after injury. Skeletal muscle injuries occur frequently in daily life and exercise, and the capacity of regeneration is critical for the repair and functional maintenance of skeletal muscle. The regeneration of adult muscle is based on the activation of satellite cells (SCs), which are mononuclear progenitors of skeletal muscle and are located between the sarcolemma and basal lamina [1]. After injury, the regeneration of muscle occurs in three overlapping stages: in the first stage, inflammatory cells infiltrate into damaged sites, and necrotic fiber fragments are removed; in the second stage, SCs are activated and proliferate into myoblasts, thereafter differentiating and fusing to form new muscle cells and replace damaged fibers; the last stage involves the maturation of newly formed fibers and the remodeling of damaged muscle [2,3]. The processes of regeneration are highly coordinated, and the expression of genes involved in regeneration are spatially and temporally regulated [4,5]. Numerous studies have been conducted to investigate the molecular mechanisms underlying muscle regeneration. A comprehensive understanding of the events involved in muscle regeneration will facilitate the treatment of skeletal muscle diseases.

In order to achieve a better understanding of muscle regeneration following physiological injury, the innervation, tendons, vascularization, and SCs should not be injured in mouse models because they contribute to myogenesis following injury. Cardiotoxin (CTX), derived from *Naja pallida*, induces a transient and reproducible acute injury without affecting the vasculature or nerves, and then produces a consistent injury in the whole muscle followed by synchronized regeneration [6,7,8]. Its application also has the advantages of allowing molecular and biochemical analyses to be performed on the whole muscle in contrast to physiological injury models induced by exercise [9,10]. Additionally, CTX injury models have relatively low harmfulness for animals compared with other non-physiological models such as crushing models [11]. Due to these characteristics, the CTX-induced skeletal muscle injury model is a suitable model for exploring the mechanisms of skeletal muscle regeneration.

In this review, we summarize the effects and mechanisms of different mouse models for obesity, diabetes, exercise training, and nutrition on regeneration after CTX-induced muscle injuries. The results may provide therapeutic targets for the repair of damaged muscle in addition to new ideas for further studies.

## 2. The Characteristics and Positions of Injury in CTX-Induced Skeletal Muscle Injury Models

CTX, a natural amphiphilic peptide derived from *Naja pallida*, can affect membrane calcium binding sites, and lower the threshold of calcium-modulated calcium ion release from the sarcoplasmic reticulum, thereafter inducing the destruction of skeletal muscle [12,13]. Muscle injury occurs at days 1 to 2 after CTX injection, where inflammatory cells infiltrate and SCs are activated to proliferate; at days 3–5, the myoblasts are induced to differentiate; at days 5–7, the new fibers with a central nucleus begin to form; and at days 10–14, the major muscle structures are restored; at day 28, the damaged muscles have almost completely recovered [14,15,16]. Due to its characteristics of transience and reproducibility, the CTX-induced injury model has been widely used to explore the mechanisms of skeletal muscle regeneration.

In CTX-induced injury models, the damaged sites are hindlimb muscles, where injuries also often occur in humans. In the related literature, the tibialis anterior is the most widely studied site in CTX-induced injury models. This is because of its obvious location and the characteristics of having a mixture of fiber types. Additionally, as a highly heterogenous muscle, tibialis anterior has only one belly, which results in uniform injury. Gastrocnemius consists mainly of fast-twitch fibers and is a bicep muscle, which may result in nonuniform injury despite the obvious location. Furthermore, other hindlimb muscles were also used in the studies such as the extensor digitorum longus, soleus, and quadriceps. The characteristics of only one type of muscle fiber and the muscle group may lead to a preference for position.

Notably, there are still some limitations in CTX-induced skeletal muscle injury models. First, the skeletal muscles include antigravity (e.g., gastrocnemius, quadriceps) and non-antigravity muscles (e.g., tibialis anterior, biceps brachii) [17]. The mechanisms identified in CTX-induced non-antigravity muscle injury models may not apply directly to the CTX-induced antigravity muscles. Second, in CTX-induced injury models, it always does not affect the vasculature or nerves in muscles [8]. In contrast, the vasculature or nerve damage often occurs during the pathogenesis of human muscle injuries [18]. This discrepancy limits the exploration of the contribution of vasculature or nerves in muscle regeneration using CTX-induced muscle injury models. Third, CTX may induce a complete necrosis of the small muscles such as EDL when examined in cross-section 48 h after injection [19]. This may make it impossible to explore the mechanisms involved in the early stages of these muscles.

## 3. Skeletal Muscle Regeneration in Different Mouse Models after CTX-Induced Skeletal Muscle Injury

CTX has been used to induce skeletal muscle injury in many mouse models (Table 1) including that of diabetes, obesity, aging, exercise training, mechanical loading, and nutrition intervention, among others. Studies have shown that streptozocin and gene mutation-induced diabetes [20,21,22,23], high fat diet-induced obesity and ob/ob mice [22,24,25], cancer cachexia [26], aging [27,28], irradiation [29], elevated carbon dioxide (CO_2_) level [30], and hindlimb suspension [31,32] lead to impaired regeneration, whereas exercise training [33,34], microcurrent electrical neuromuscular stimulation [35], microelement zinc [36], and overloading [37,38] improve the regeneration of CTX-induced damaged muscle. The accumulation of mitochondrial DNA alterations activates muscle regeneration in myofibers during aging, but leads to reduced muscle mass [39].

Gender and sex hormone levels also influence the regeneration processes in CTX-induced muscle injuries. Males exhibit larger newly formed fibers than females at the same age after injury, whereas females show higher fat deposition than males during regeneration [40,44] and also remove necrotic tissue more rapidly [44]. Castration of males increases the cross-sectional areas (CSAs) of the newly formed fibers and fat accumulation, whereas ovariectomized mice exhibit inhibited regeneration and decreased adipocyte accumulation, and estrogen supplementation rescues regeneration in ovariectomized mice [41,44]. Lack of estrogen-related receptor α also impairs the recovery of mitochondrial energetic capacity and decreases the activity of adenosine 5′-moophpsphate (AMP)–activated protein kinase (AMPK), which then also leads to delayed regeneration [52].

Additionally, the studies also revealed that different mouse strains have similar regeneration processes with no significant morphological and functional differences. However, the mechanisms of skeletal muscle regeneration may be strain-dependent. For instance, toll-like receptor 4 (TLR4) plays distinct roles in the injured muscle of C57BL/6 and C3H/HeJ [49,53].

It was also reported that the regeneration of skeletal muscle is position-specific: after CTX injury in tibialis anterior and the masseter, head muscles recover slowly and eventually return to the base level, whereas limb muscles show quicker recovery and eventually excessive growth [50].

## 4. Mechanisms of Regeneration in CTX-Induced Injury Models

It has reported that the trajectories of skeletal muscle regeneration vary considerably despite achieving complete regeneration in different injury models [54], wherein the mechanisms of regeneration in damaged skeletal muscle depend on the injury models [54]. In the following section, we summarize the mechanisms of regeneration based on CTX-induced skeletal muscle injury.

### 4.1. Inflammatory Response in CTX-Induced Injury Models

Inflammatory response could play an important role in timely skeletal muscle regeneration after CTX-induced injury. In this section, we summarize the mechanisms of this process and its three stages: immune cell infiltration, M1 to M2 macrophage polarization, and the clearance of necrotic fiber debris. 

#### 4.1.1. The Mechanisms of Inflammatory Response in CTX-Induced Skeletal Muscle Injury

**Figure 1:**
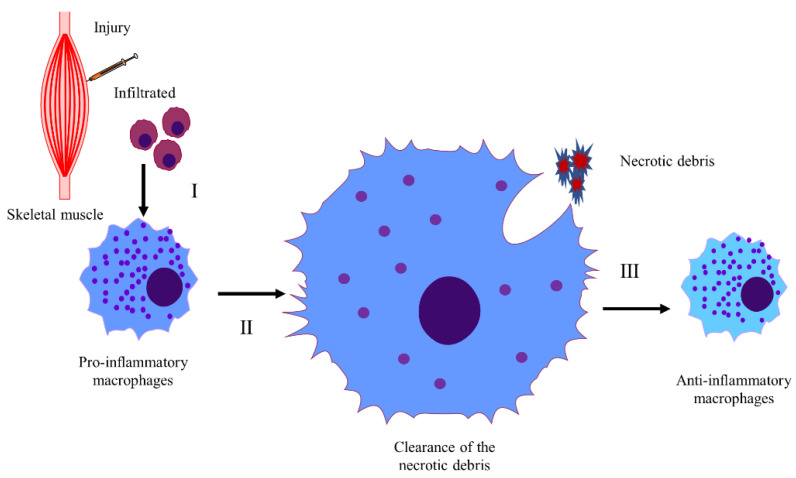
The inflammatory response in CTX-induced skeletal muscle injury. (**I**) Infiltration of the immune cells. Upon injury, the immune cells are recruited into the damaged area, then induce inflammatory response in the damaged muscle. In this process, the lack of Klf2, STAT1, Hmox1, MKP-1 and upregulation of adiponectin (APN), activin A, and calmodulin (CaM) signaling increase, while the lack of IL-1a/β, Hsp70, CCR2, and IL-6/STAT3 and so on, inhibits the inflammatory infiltration. (**II**) Elimination of necrotic muscle fibers. The monocyte infiltrated into the damaged site, and become pro-inflammatory macrophages (M1 macrophages). M1 macrophages could secrete proinflammatory cytokines, maintain the inflammatory environment, then clean the necrotic debris. In this process, RetSat/MFG-E8 is required in the regulation of efferocytosis. ADAM8/PSGL-1 signaling, the TAM kinase signaling pathway, and the expression of SRB1 facilitate the elimination of necrotic fibers. Additionally, supplementation of balenine also increases the phagocytosis ability of macrophages. (**III**) Polarization of the macrophages. As the clearance of the muscle debris, the pro-inflammatory macrophages switch to anti-inflammatory macrophages (M2 macrophages), then secrete anti-inflammatory factors and stimulate regeneration. TG2 deficiency and the excessive calmodulin-dependent signaling delay/impair, while PGC-1α, SRB1, and so on, stimulate the polarization of the macrophages.

Upon injury, immune cells residing in the skeletal muscle are rapidly activated and then release tissue destruction factors to accelerate muscle injury [55]. Additionally, the immune cells also secrete cytokines such as tumor necrosis factor alpha (TNFα) and interleukin 6 (IL-6), recruiting neutrophils into damaged areas, which in turn stimulates the secretion of chemokines including monocyte chemoattractant protein 1 (MCP-1), macrophage inflammatory protein 1 alpha (MIP-1α), MIP-1β, and promotes the invasion of circulating monocytes [56]. Studies have shown that the mechanisms of inflammatory response involved in CTX-induced skeletal muscle regeneration are complex (Figure 1, Table 2). 

It is reported that the lack of interleukin (IL-1) [77], CC chemokine receptor 2 (CCR2) [81], toll-like receptor 2 (TLR2) [79], and heat shock protein (Hsp70) [78] and the inactivation of IL-6/signal transducer and activator of transcription 3 (STAT3) signaling [82] and complement C3a-C3a receptor (C3aR)/CCL5 signaling [86] lead to reduced/delayed monocyte/macrophage infiltration, which then reduces the clearance of necrotic fiber debris and impairs myoblast proliferation, attenuating/delaying muscle regeneration. The endogenous conversion of n-6 to n-3 polyunsaturated fatty acids [76] and pretreatment with thymol [75] reduce macrophage infiltration and cell apoptosis, leading to increased SC migration and proliferation and improved muscle regeneration. Loss of Kruppel-like factor 2 (KLF2) [65], and the lack of plasminogen activator inhibitor (PAI-1) [2,23] and signal transducer and activator of transcription 1 (STAT1) in bone marrow-derived cells [66] and the inhibition of activin A [69] stimulate monocyte/macrophage recruitment, accelerate damaged muscle degradation, and promote myoblast proliferation, thereby improving muscle regeneration. Additionally, the accumulation of interleukin 17A (IL-17A)-producing T cells can also promote muscle regeneration in a microbiota-dependent way [97]. Lack of neuraminidase-1 (Neu1) [58] and estrogen signaling [59] and increased activation of calcium/calmodulin-dependent protein kinase IV (CaMKIV) [57] increase the inflammatory response but inhibit muscle regeneration. This may be because the lack of Neu1 leads to delayed myoblast differentiation and myofiber maturation [58]; however, the activation of CaMKIV and the lack of estrogen signaling increase the infiltration of pro-inflammatory macrophages, impair the transition of macrophages from M1 to M2, reduce the phagocytosis of macrophages, resulting in impaired muscle regeneration [57].

In addition, muscle cells are also involved in the immune response. Studies have shown that the inflammatory environment induced by interferon gamma (IFN-γ) stimulates the expression of major histocompatibility complex (MHC) and some co-stimulatory molecules from regenerated myofibers or cultured myoblasts and myotubes, which then contribute to the immune response [98]. Myofibers also mediate the inflammatory response through the activation of transforming growth factor beta (TGF-β)/IL-6 signaling, and direct Th17 and Treg cell responses [99]. Moreover, oxidative stress during the inflammatory response can also change the structure and function of proteins, which then regulates muscle regeneration in CTX-induced injury [100].

#### 4.1.2. The Mechanism of Macrophage Polarization in CTX-Induced Skeletal Muscle Injury

Macrophages are the main inflammatory cells, and macrophage polarization is involved in the regulation of regeneration [79]. Infiltrated monocytes differentiate into pro-inflammatory M1 macrophages, secreting proinflammatory cytokines, cleaning up necrotic fiber debris, and maintaining an inflammatory environment. Upon the removal of necrotic fiber debris, M1 macrophages switch to M2 macrophages, secreting anti-inflammatory factors and stimulating regeneration [101]. Research shows that (Figure 1, Table 2) a preexisting inflammatory environment [91], irradiation [29], transglutaminase 2 (TG2) deficiency [102], and the excessive activation of calmodulin-dependent signaling [64] delay or impair the M1 to M2 macrophage conversion, which then delays or impairs muscle regeneration. On the other hand, estrogen signaling [59], extracellular vesicles (EVs) derived from mesenchymal stem cells (MSCs) [73], peroxisome proliferator-activated receptor γ coactivator 1α (PGC-1α) [56], and scavenger receptor class B1 (SRB1) [90] stimulate macrophage polarization, eliminate necrotic fibers, reduce fibrosis, and then induce muscle regeneration. In addition, the lack of progranulin prolongs the existence of M2 macrophages and increases the size of newly formed fibers [72]. Increased activation of peroxisome proliferator-activated receptor beta (PPARβ) promotes the recruitment of M2 macrophages and accelerates the regeneration processes [70].

#### 4.1.3. The Mechanism of Necrotic Fiber Debris Clearance in CTX-Induced Skeletal Muscle Injury

The phagocytotic ability of macrophages plays an important role in the elimination of necrotic fiber debris (Figure 1). The lack of retinol saturase (RetSat) in macrophages results in less milk fat globule-epidermal growth factor-factor-8 (MFG-E8) produced and impaired efferocytosis [103]. However, skeletal muscle regeneration in RetSat-null mice is normal following CTX-injury. This is because other cell types participating in muscle regeneration such as myoblasts compensate for the impaired macrophage function, which leads to normal muscle regeneration in RetSat-null mice [93]. Supplementation with balenine promotes the infiltration of immune cells into damaged muscle, and increases the phagocytotic ability of macrophages, leading to improved regeneration [74].

Additionally, the genes involved in the clearance of necrotic muscle fiber debris are highly expressed in immune cells (Table 2). Disintergrin and metalloprotease (ADAM) 8 expression in neutrophils reduces the expression of P-selectin glycoprotein ligand-1 (PSGL-1) on the surface of neutrophils, which then increases the ability of neutrophils to infiltrate into damaged areas and contribute to the removal of fiber debris [88]. Tyro3/Axl/Mer (TAM) kinase signaling mediated by Axl/Mer (AM) receptor Mer expressed in CD45+ cells and SRB1 expressed in macrophages could also facilitate the elimination of necrotic fibers and stimulate macrophage transition from M1 to M2 [89,90].

### 4.2. SC Activation and Myoblast Proliferation, Differentiation, and Fusion in CTX-Induced Injury Models

Upon injury, the regeneration capacity of the skeletal muscle is due to the SCs, and the critical steps such as SC activation and myoblast proliferation, differentiation, and fusion determine the extent of regeneration. Additionally, myotube maturation and the self-renewal of SCs also influence regeneration. 

#### 4.2.1. Mechanisms of SCs Activation and Myoblast Proliferation in CTX-Induced Injury Models

**Figure 2:**
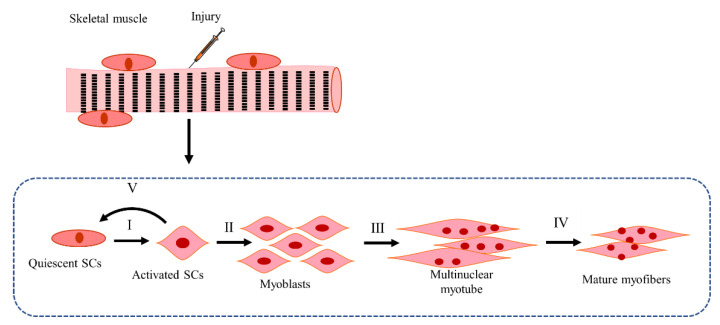
SC activation, myoblast proliferation, differentiation and fusion, and the myotube maturation in CTX-induced injury models. (**I**) SC activation. In the damaged muscle, the quiescent SCs are activated to repair the skeletal muscle. Conversion of n-6 to n-3 polyunsaturated fatty acids (PUFAs), Salvador, testosterone, upregulation of Insl6, and so on, promote, while the lack of Nrf2, LCN2, and miRNA-501 inhibit the activation of SCs. (**II**) Myoblast proliferation. Activated SCs begin to proliferate and become myoblast. FGF2, RING finger protein13 (RNF13) derived from macrophages, Sod1/Cat, lack of p21, and so on facilitate, while FOXO1/p57/Gadd45α and CaMKK2/AMPK, lack of Gαi2 and H19/Igf2, etc., inhibit myoblast proliferation. (**III**) Myoblast differentiation and fusion. The expended myoblasts experience differentiation and fuse with other muscle fibers to form multinuclear myotubes. Lack of SLK and APOBEC2, upregulation of miR-206, miR-378, MMP-13, and Ccndbp1 enhance; Parkin, tension, Rbm24, and Hsp70 deficiency inhibit the capacity of myoblast differentiation. Lack of ANO5, HMGR, BAI3, and miR-188, and so on, inhibit, upregulation of Nox4/ROS, PLD1, Mustn1, and TRPV1 stimulate myoblast fusion. (**IV**) Maturation of myofibers. Lack of Dcx, PTEN, and desmin influence the myofiber maturation. (**V**) Self-renewal of SCs. The activated SCs proliferate to replenish the SC pool of the skeletal muscle. In this process, Angiotensin II, lack of mTORC2, AIF, SelN, and Notch1/Notch2 impair, while Lgr5, laminin-111, lack of PKC θ, Rb1, and Pax7 acetylation stimulate SC self-renewal or SC pool replenishment.

In normal adult muscle, SCs are in a quiescent state (Figure 2) and express paired box 7 (Pax7); once injury occurs, SCs begin to express myogenic differentiation 1 (MyoD) and myogenic factor 5 (Myf5) and are activated and then enter the cell cycle [104,105,106]. During this process, the basement membrane plays an important role in triggering the activation of SCs. After injury, the components of the basement membrane that mediate the contacts of the basal lamina to SCs and myofibers are degraded, and key components of the basement membrane (collagen IV alpha 1, laminin gamma-1, nidogen-2, and heparane sulfate proteoglycan-2) are downregulated, which further leads to a release of growth factors from the dismantling basement membrane and increased the elasticity of the SC niche, thus providing a suitable environment for SC proliferation [107]. As shown in Table 3, Xin and insulin-like 6 (Insl6) are involved in the activation of SCs. Insl6 overexpression in muscle facilitates SC activation and proliferation through the reduction in cell apoptosis upon CTX injury [108]. Xin, which increased in SCs within 12 h following the CTX-induced injury, maintains the activation of SCs, and the downregulation of endogenous Xin leads to the increased proliferation and migration of myoblasts [109]. Other research has shown that Xin deficiency reduces the activation and proliferation of SCs, and muscle regeneration is then impaired through the reduction in primary myoblasts and increased apoptosis of SCs [110,111]. Additionally, studies have also reported that the over-activation of myostatin/TGF-β receptor/pSmad3 signaling in diabetic mice [43] inhibits the activation of SCs. Nevertheless, the inhibition of TGF-β signaling by simultaneous knockout of TGF-β type I receptor (Tgfbr1) and activin receptor type 2B (Acvr1b) accelerates the myogenic process and improves skeletal muscle regeneration [112], whereas knockout of TGF-β receptor II (TGF-βr2) increases the inflammatory response by affecting T-cell function and the withdrawal at the later stage of muscle regeneration. The lack of nuclear factor (erythroid-derived) like 2 (Nrf2) [113] and lipocalin-2 (LCN2) [114] also inhibits the activation of SCs. These may be associated with the pro-oxidation state, reactive oxygen species (ROS) accumulation, and reduced matrix metalloproteinase-9 (MMP-9) expression, which then lead to delayed or impaired regeneration [113,114]. 

The capacity for myoblast proliferation is influenced by numerous cytokines. Pretreatment with acetylated myostatin 1 (Ac-MIF1) and acetylated and amidated myostatin 2 (Ac-MIF2-NH2) stimulates muscle regeneration by increasing the capacity of myoblasts for proliferation and differentiation [242]. Chemokines including MCP-1, MIP-1α, or MIP-1β (CCL4) induce extracellular regulated protein kinase (ERK) 1/2 phosphorylation through a Gαi subunit-dependent manner, which promotes myoblast proliferation [170]. Gαi2 also promotes myoblast differentiation through the protein kinase C (PKC)/glycogen synthase kinase 3β(GSK3β)/miR-1 pathway or the histone deacetylase (HDAC) inhibition [160]. Nitric oxide (NO) stimulates the proliferation of SCs via a cyclic guanosine 3′,5′-monophosphate (GMP)-dependent pathway [145]. Double homeobox gene (Duxbl) [151] and factor for adipocyte differentiation 24 (fad24) [172] promote myoblast proliferation via increasing the cell cycle. The increased expression of anti-oxidant superoxide dismutase 1 (Sod1) and catalase (Cat) genes also facilitates the potential of proliferation and differentiation [153]. Additionally, the lack of H19 [169], hippo inhibition [117], and lack of heme oxygenase-1 (Hmox1) [67], fibroblast growth factor 6 (FGF6) [157], p38α, and p38γ [148] have also been found to promote myoblast proliferation, although the mechanisms remain unknown. Tweak/Fn14 also contributes to myoblast proliferation but inhibits their differentiation and delays their regeneration [168]. In contrast, increased inflammation, cell cycle inhibition, the destruction of membrane integrity, and iron accumulation all lead to attenuated myoblast proliferation. The results show that the lack of mitogen-activated protein kinase phosphatase-1 (MKP-1) [63] and heat shock transcription factor 1 (HSF1) [128] increased the inflammation and secretion of proinflammatory cytokines, the lack of α7 integrin destroyed the sarcolemmal integrity [165], and impaired fibroblast growth factor (FGF) responsiveness induced by deficiency of Cdon or fibroblast growth factor receptor 1 (FGFR1) led to the impairment of myoblast proliferation [164,174]. Lack of early growth response3 (Egr3) and the overexpression of calcium/calmodulin-dependent protein kinase kinase 2 (CaMKK2) might induce cell cycle arrest by the inactivation of nuclear factor kappa-B (NF-κB) and the activation of AMPK/p-cdc2-Tyr15 signaling, respectively [159,171]. Peroxisome proliferator-activated receptor δ (PPARδ) deficiency reduced forkhead box protein O1 (FOXO1) expression, which then impaired proliferation; FOXO1 overexpression also induced the expression of cell cycle inhibitors p57 and Gadd45α, which decreased the capacity for myoblast proliferation [127,167]. Iron accumulation and ROS production induced by transferrin receptor 1 (Tfr1) deletion also led to defective myoblast proliferation via the Tfr1–Slc39a14–iron axis [162]. Additionally, the deletion of Notch1 and/or Notch2 [141] and lack of nuclear T3 receptor TRα1 (p43) [158] also inhibited myoblast proliferation. However, the lack of Nur77 did not impair muscle regeneration even though it inhibited myoblast proliferation [150].

MicroRNAs are important regulators in SC activation and myoblast proliferation. The overexpression of miR-378 attenuates the activation and differentiation of SCs in an insulin-like growth factor 1 receptor (IGF1R)-dependent manner, which then delays the regeneration [118]. The expression of miR-29a, induced by fibroblast growth factor 2 (FGF2), reduces the expression of basement membrane members, which results in dismantling of the basement membrane, further providing a suitable environment for myoblast proliferation [107].

Epigenetic regulation is also involved in muscle regeneration. DNA methyltransferases 3a (Dnmt3a) ablation in SCs leads to hypomethylation of p57Kip2, which further induces a higher expression of p57Kip2, and then impairs the SC proliferation and attenuates the regeneration of damaged skeletal muscle [163]. Mixed lineage leukemia protein-1 (MLL1) facilitates the proliferation of myoblasts and Pax7+ SCs by epigenetically increasing the expression of Myf5 by mediating the trimethylation of lysine 4 on the histone H3 protein subunit (H3K4me3) enriched on the Myf5 promoter [123].

#### 4.2.2. Mechanisms of Self-Renewal of SC Pool in CTX-Induced Injury Models

During regeneration, the self-renewal of SCs is also essential for the repair of damaged muscle (Figure 2). The activated SCs downregulate MyoD expression, and then replenish the SC pool through both symmetric cell division and asymmetric cell division [104,143]. In this process, primary cilium, harbored on the surface of quiescent SCs, has been shown to be an intrinsic factor controlling the self-renewal of SCs. Upon SC activation, primary cilia disassemble, and SCs enter the cell cycle. Upon exit from the cell cycle, the primary cilia reassemble again at the surface of a minority of SCs that are committed to self-renewal [143]. Disruption of the cilia reassembly impairs the self-renewal of SCs [143]. Additionally, the lack of selenoprotein N (SelN) [140], angiotensin II/Ang II AT1 receptor (AT1R) [137], and thyroid hormone receptor alpha (TRa) deficiency [139] resulted in a reduced SC pool and impaired regeneration of damaged muscle. Mammalian target of rapamycin complex 2 (mTORC2) depletion does not affect the myogenic function of SCs but impairs the replenishment of the SC pool upon repeated CTX injury [136]. Lack of collagen VI reduces the self-renewal capacity of SCs and impairs muscle regeneration [146].

In contrast, using the CRISPR/Cas9 mutagenesis technique, Sincennes et al. abolished Pax7 acetylation in mice and demonstrated that the lack of Pax7 acetylation led to reduced numbers of asymmetric stem cell divisions, expansion of the SC pool, and increased numbers of oxidative II A myofibers [124]. PKCθ deficiency upregulates Pax7 expression and activates Notch signaling for the maintenance of the self-renewal capacity of SCs in CTX-injured mdx mice [121]. Retinoblastoma (Rb) ablation in SCs increases the cell cycle re-entry of quiescent SCs and promotes the expansion of SCs. However, sustained retinoblastoma 1 (Rb1) loss impairs muscle fiber formation [135]. In addition, Klotho rejuvenates aged SCs and maintains the function of SCs by inhibiting the Wnt signaling pathway [129].

#### 4.2.3. Mechanisms of Myoblast Differentiation in CTX-Induced Injury Models

The mechanisms of myoblast differentiation in Figure 2 and Table 3 show that the upregulation of myogenic regulatory factors (MRFs) such as MyoD and myogenin is associated with the increased capacity of myoblast differentiation, which further contributes to skeletal muscle regeneration in CTX-induced injury models. Research suggests that A-kinase anchoring protein 6 (AKAP6) [195], andrographolide [218], mouse double minute 2 homolog (Mdm2)/CCAAT/enhancer-binding protein β (C/EBPβ) [214], apolipoprotein B mRNA editing enzyme catalytic polypeptide 2 (APOBEC2) knockout [8] induces the expression of MyoD, myogenin, MyoG, and desmin. Leucine-rich repeats and transmembrane domains 1 (LRTM1) inhibit the recruitment of p52Shc to FGFR1 and inhibit the activation of ERK, further reducing the inhibition of cyclin dependent kinase 4 (CDK4) on the transcriptional activity of MyoD [196]. The increased MyoD interacts with its targets transcription elongation factor A-like 7 (Tceal7) and R3h domain containing-like (R3hdml) and promotes myoblast differentiation [1]. Additionally, cyclin D-type binding-protein 1 (Ccndbp1) can bind to MyoD and regulate muscle differentiation [3,186]. The energy metabolism in cells also influences myoblast differentiation. Micropeptide in mitochondria (MPM) increases oxygen consumption and adenosine triphosphate (ATP) synthesis and promotes myoblast differentiation [197]. The expression of the type 1 canonical subfamily of transient receptor potential channels (Trpc1) promotes the influx of calcium in myoblasts during differentiation and activates the phosphatidylinositol-3-kinase (PI3K)/AKT/mTOR/p70S6K pathway [173]. The decrease in chondroitin sulfate (CS) also stimulates the activation of PI3K/AKT signaling [181], which leads to faster regeneration [243]. Additionally, trimetazidine modulates the metabolic shift from free fatty acid β-oxidation to glucose oxidation by stimulating AMPK/PGC1α, and inducing autophagy, both of which contribute to myoblast differentiation [179]. Furthermore, the lack of signal transducer and activator of transcription 6 (STAT6) increases myoblast differentiation in an IL-4-independent way [219]. Angiotensin type 2 receptor (AT2R) inhibits the activation of ERK1/2 signaling to promote myoblast differentiation and fusion [185]. Inositol requiring enzyme 1 (IRE1) suppresses the expression of myostatin through its RNase-dependent RIDD activity, which then promotes the differentiation of myoblasts [188].

In contrast, the increased oxidation state impairs myoblast differentiation. Nonalcoholic fatty liver disease (NAFLD) reduces the SC pool and impairs SC differentiation, leading to attenuated skeletal muscle regeneration. This may be associated with TNF-α upregulation and increased levels of oxidative stress marker nicotinamide adenine dinucleotide phosphate (NADPH) oxidase-2 (NOX 2) [47]. The inhibition of carbonyl reductase1 (CBR1) leads to increased ROS levels and diminishes myoblast differentiation [216]. Iron overload impairs myoblast differentiation through oxidative stress-induced inactivation of the mitogen-activated protein kinase (MAPK) signaling pathway [45]. Lack of Hsp70 [16] and TNF-α receptors p55 and p75 [187,209] also downregulate p38MAPK activation, which further impairs myoblast differentiation. Moreover, decreased expression of MRFs such as MyoD and myogenin also impairs differentiation. Overexpression of ladybird homeobox 1 (Lbx1) [213] and the tripartite motif domain of myospryn [203] inhibit the expression of MyoD and myogenin. High levels of cardiotrophin-1 (CT-1) repress the expression of the MRFs such as MyoD through the activation of mitogen-activated protein kinase kinase (MEK)-MAPK signaling [199]. Protein-activated kinase 1 (PAK1) inhibitor IPA-3 decreases the expression of myogenin and reduces p38 phosphorylation [193]. Teashirt-3 (Tshz3) cooperates with BRG1-associated factor 57 (BAF57) and inhibits the MYOD-dependent activation of Myog [201]. Additionally, HS 6-O-endosulfatases (Sulfs) mutation and lack of tensin lead to reduced withdrawal from the cell cycle and delayed myoblast differentiation [191,211]. The deletion of RNA binding motif protein 24 (Rbm24) also regulates the alternative splicing of myogenic associated genes such as myocyte enhancer factor 2d (Mef2d), Rho-associated protein kinase 2 (Rock2), further inhibiting myoblast differentiation [106].

MicroRNAs are involved in the regulation of myoblast differentiation. The overexpression of miR-351 protects differentiating myoblasts from apoptosis by regulating the target gene E2f3 and contributes to myoblast differentiation [152]. In a normoxic state, miR-210 induces myoblast differentiation in a hypoxia-inducible factor 1-α (Hif1a)-dependent manner [239]. Overexpression of Linc-smad7 increases the expression of smad7 and insulin-like growth factor 2 (IGF2), which then induces myoblast differentiation [178]. In addition, miR-431 directly interacts with the 3′ untranslated region of Smad4. Ectopic miR-431 injection greatly reduces Smad4 levels and improves muscle regeneration in CTX-induced skeletal muscle injury models, whereas the inhibition of miR-431 significantly represses myoblast differentiation [192]. The inhibition of miR-188 reduces the expression of myogenic regulator factor 4 (MRF4) and Mef2c and impairs myoblast differentiation, whereas the overexpression of miR-188 has the opposite effect [155]. Knockdown of transactivating response RNA-binding protein (Trbp) downregulates the expression of miR-1a and miR-133a and reduces myotube formation [244]. Intriguingly, MyoR, a muscle-restricted basic helix–loop–helix transcription factor that antagonizes the actions of MyoD, is found to be anticorrelated with miR-378 during CTX-induced muscle regeneration. MyoD binds to the miR-378 gene and causes both transactivation and chromatin remodeling, thus upregulating miR-378 during myogenic differentiation. The 3′ untranslated region of MyoR contains a direct binding site for miR-378. The presence of this binding site significantly reduces the ability of MyoR and prevents the MyoD-driven transdifferentiation of fibroblasts [180].

Epigenetic regulation is also involved in myoblast differentiation. Histone- and protein arginine methyl transferases 5 (PRMT5)-associated protein COPR5 is required for cell cycle exit and myoblast differentiation. The silencing of COPR5 reduces PRMT5 recruitment to the promoters of p21 and MYOG by hindering interaction with the Runt-related transcription factor 1 (RUNX1)-core binding factor-β (CBFβ), which then inhibits the expression of p21 and MYOG and further impairs myoblast differentiation [200]. IGF-1 induces the phosphorylation and activation of ATP citrate lyase (ACL) through the PI3K/AKT pathway. The activated ACL catalyzes the conversion of citrate into oxaloacetate and acetyl-CoA, and acetyl-CoA can be further utilized by histone acetylases to acetylate H3 (K9/14) and H3 (K27) at the MyoD locus to increase MyoD expression, thereby promoting myoblast differentiation [184].

#### 4.2.4. Mechanisms of Myoblast Fusion in CTX-Induced Injury Models

Differentiated myoblasts fuse with damaged fibers or new myotubes by cell–cell recognition, adhesion, migration, and membrane fusion, subsequently forming multinucleated myotubes [106,230]. This is a dynamic and coordinated process involving many proteins (Figure 2, Table 3).

In terms of stimulating fusion, anoctamin 5 (ANO5) stimulates the repair of the sarcolemmal membrane and facilitates myoblast fusion [224]. Stabilin-2 activates the G-protein coupled receptor (GPCR) activity of BAI3 and then recruits Elmo to the membrane to stimulate myoblast fusion [226]. NADPH oxidase 4 (Nox4) induces the expression of myomarker fusion protein (Tmeme8c) via Nox4-mediated ROS production and then contributes to myoblast fusion [229]. The activation of phospholipase D1 (PLD1) on the plasma membrane facilitates mononucleated myoblast fusion with nascent myotubes [230]. Inhibition of the hierarchical non-clustered miRNA network including highly active (miR-29a), moderately active (let-7), and mildly active (miR-125b, miR-199a, miR-221) networks, stimulates the activation of focal adhesion kinase and AKT and MAPK signaling, and leads to the formation of myotubes [189]. Transient receptor potential cation channel vanilloid I (TRPV I) can be activated by IL-4 and calcium signaling, which then facilitates myoblast fusion instead of proliferation [232]. Syncytin contributes to myoblast fusion, and this effect is male-specific [222].

The mechanism of inhibition of myoblast fusion involves the upregulation of TGF-β via calpain-3 (CAPN3) deficiency, thus leading to defective myoblast fusion [234]. C1q-like 1-4 interacts with BAI3 to repress myoblast fusion [226]. Lack of IGF-1 receptor (IGF-1R) signaling leads to reduced fiber fusion via growth hormone receptor-independent signaling [220]. The expression of (Pro)renin receptor ((P)RR) activates the Wnt/β-catenin and Yes-associated protein (YAP) signaling pathways, and decreases myoblast fusion [228]. In addition, transglutaminase 2 (TG2) [102], constitutive expression of c-Myb lacking its 3′ untranslated region (3′ UTR) [223], inhibition of 3-hydroxy 3-methylglutaryl coenzyme A reductase (HMGR) [225], myasthenia gravis [46], and the lack of ste20-like kinase (SLK) [221] also impair the capacity of myoblast fusion and decrease the fusion index.

#### 4.2.5. Mechanisms of Myotube Maturation in CTX-Induced Injury Models

Fused multinucleated myotubes undergo terminal differentiation and eventually become mature myofibers. Kruppel-like factor (Klf5) (Table 3) is shown to interact with MyoD and Mef2 to regulate terminal differentiation [3]. Doublecortin (Dcx) facilitates myofiber maturation [235]. Sema4C stimulates the phosphorylation of p38 and activates the p38/MAPK signaling pathway to promote terminal differentiation [176]. The expression of clathrin heavy chain like 1 (CHC22) in CTX-induced injury muscle, however, diminishes glucose transporter 4 (Glut4) response and further impairs fiber maturation [237].

### 4.3. Fibrosis in CTX-Induced Injury Models

**Figure 3:**
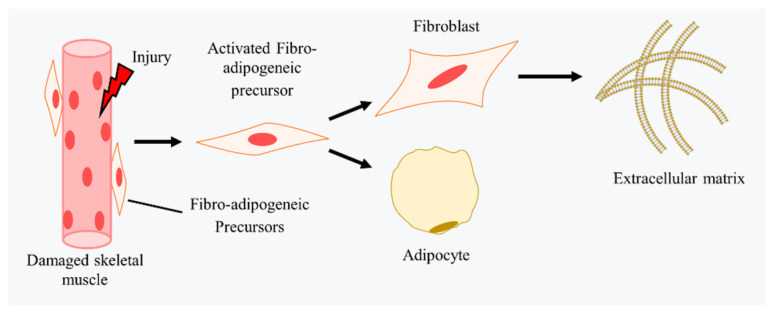
Fibrosis in CTX-induced injury models. After injury, FAPs are activated and begin to proliferate and then differentiate into fibroblasts and adipocytes. Fibroblasts secrete extracellular matrix proteins and growth factors, and differentiate into myofibroblasts to increase α-SMA expression and ECM synthesis, which may provide scaffolds for the skeletal muscle regeneration while abnormal deposition of ECM finally results in fibrosis. In CTX-induced injury muscle, increases in miR-199a-5p, growth differentiation factor 11 (GDF11), platelet-derived growth factor receptor beta (PDGFRβ), lack of GDF-associated serum protein-1 (Gasp1) and/or Gasp2, and a prior burst of double homeobox 4 (DUX40) induce the deposition of collagen and contribute to fibrosis. In contrast, laminin-111 and Losartam therapy reduce fibrosis. Additionally, IL-4/IL-13 signaling, EGF and BTC, and the RA signaling pathway also regulate the fate of FAPs in skeletal muscle regeneration.

Fibrosis is an important stage for regeneration (Figure 3). In this process, the temporary extracellular matrix (ECM) components serve as a scaffold for new fibers and stabilize muscle tissue [89]. In damaged skeletal muscle, fibro/adipogenic progenitors (FAPs) are considered as the main source of fibroblasts [245]. After injury, FAPs are activated and begin to proliferate. This increases FAPs in the necrotic area, which need to be removed in time. Failure to clear FAPs will result in their differentiation into fibroblasts and adipocytes [246]. Fibroblasts secrete extracellular matrix proteins and growth factors and then differentiate into myofibroblasts to increase α-smooth muscle actin (α-SMA) expression and ECM synthesis, finally resulting in fibrosis [247]. Studies on the regulation of FAPs show that (Table 4) IL-4 secreted by infiltrated eosinophils stimulates the activation of FAPs in an IL-4-dependent way. IL-4/IL-13 signaling in FAPs contributes to proliferation and adipogenic differentiation of FAPs is inhibited to facilitate regeneration [55]. IL-1α and IL-1β inhibit the adipogenic differentiation of FAPs, and epidermal growth factor (EGF) and betacellulin (BTC) stimulate the proliferation of FAPs [248]. Lack of TGF-β1 in macrophages inhibits FAP proliferation and reduces fibrosis [249]. Inactivation of retinoic acid (RA) signaling in FAPs leads to adipogenic differentiation, which then impairs regeneration [246].

Additionally, studies have also shown that increases in miR-199a-5p [247], growth differentiation factor 11 (GDF11) [254], and platelet-derived growth factor receptor beta (PDGFRβ) [245], together with the lack of GDF-associated serum protein-1 (Gasp1) and/or Gasp2 [256] and a prior burst of double homeobox 4 (DUX4) [251], induced the deposition of collagen and contributed to fibrosis. In contrast, laminin-111 reduced fibrosis and facilitated skeletal muscle regeneration [165]. Losartam therapy also reduced fibrosis by inhibiting the TGF-β signaling pathway [250].

### 4.4. Calcification in CTX-Induced Injury Models

Calcification occurs after muscle injury. Under normal conditions, calcification can be resorbed. While in a pathological state, continuous calcification can induce chronic inflammation and/or loss of muscle function [264]. Studies have revealed that (Table 4) after CTX injection, Tie2-expressing endothelial precursors are the main contributor to calcification in a mouse model of dysregulated bone morphogenetic protein (BMP) signaling [258]. Moreover, the inflammatory microenvironment induced by CTX injection is also necessary for calcification in injured muscle [258]. At the early stage after injury, calcific nodules are present in mitochondria, which are mediated by cell death and can be cleared by infiltrated macrophages [257]. Additionally, calcification in damaged muscle may also occur in connection with reduced plasmin, and this is independent of its canonical fibrinolytic function [255]. The hypoxia state induced by CTX injury can induce osteogenic differentiation and mineralization of muscle resident stromal cells and further stimulate the formation of myofiber calcification [259].

### 4.5. Angiopoiesis and Neurogenesis in CTX-Induced Injury Models

In CTX-induced injury models, the capillaries are destroyed, and endothelial cells are activated to repair the skeletal muscle endothelium. It is reported that (Table 5) macrophage cells derived from bone marrow can express endothelium-related markers such as Tie2 and CD31 to promote angiogenesis [265]. Angiotensin II derived from differentiated muscle myoblasts stimulates the migration of endothelial cells, which also further facilitates angiopoiesis [266]. In contrast, CCR2 deficiency leads to decreased vascular endothelial growth factor (VEGF) production and delayed angiogenesis in injured muscle, which then impairs regeneration [81]. 

In terms of neurogenesis, M2 macrophages infiltrate damaged muscle, produce hepatocyte growth factor (HGF), and then stimulate the expression of semaphorin 3A (Sema 3A) in myoblasts to regulate the regeneration of motor innervation in injured muscle [270,271]. Pre-activation of satellite cells delays the maturation of the neuromuscular junction by reducing the expression of semaphoring (Sema) 3A and S100B [269]. Lack of desmin leads to disrupted neuromuscular connections [238].

### 4.6. Other Regeneration-Related Genes in CTX-Induced Injury Models

In addition to the mechanism described above, there are a large number of genes involved in skeletal muscle regeneration in CTX-induced injury models such as Tsukushi, Dicer, mesoderm specific transcript (Mest), filamin C, LYVE-1, and so on (Table 6). However, in these studies, the special role of these genes has not been explored. Further experiments are needed to elucidate their function.

### 4.7. Non-SC Stem Cells Regulate Regeneration in CTX-Induced Injury Muscle

**Figure 4:**
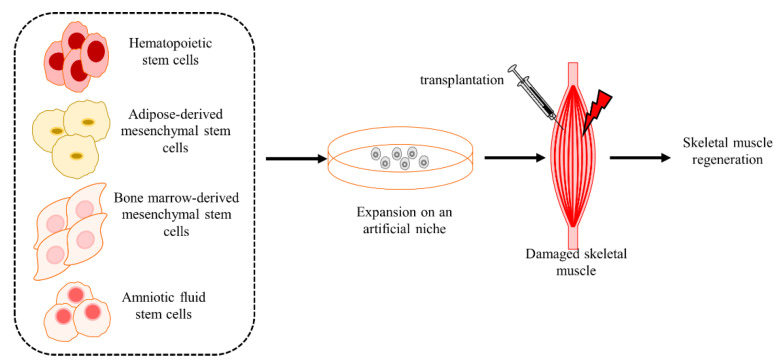
Non-SC stem cells regulate regeneration in CTX-induced injury muscle. Transplantation of hematopoietic stem cells, adipose-derived mesenchymal stem cells, bone marrow-derived mesenchymal stem cells, and amniotic fluid stem cells stimulate regeneration in the damaged skeletal muscle.

Non-SC stem cells are also involved in the regulation of regeneration (Figure 4). The results (Table 7) show that bone marrow-derived cells [308,309], pulp cells [310], bone marrow-derived human MSCs [311], hematopoietic stem cells [312], muscle precursor cells [313,314], capillary stem cells [315], adipose-derived mesenchymal stem cells [316], and human amniotic fluid stem cells [317,318] settle in the injured sites and differentiate into myogenic cells to stimulate the skeletal muscle regeneration in CTX-induced injury models. Additionally, mobilization of bone marrow stem cells also accelerates the muscle regeneration [319].

## 5. Conclusions

Skeletal muscle has a tremendous capacity for regeneration after injury. This is largely due to muscle SCs. In order to learn about the mechanisms of regeneration, skeletal muscle regeneration has been studied for decades in numerous injury models. However, differences in injury exist among the different models, which makes their comparison difficult. In the CTX-induced injury model, a transient and reproducible acute injury is induced without affecting the vasculature or nerves, and this allows for the possibility of performing molecular and biochemical analyses of the whole muscle. Additionally, CTX injury models have a relatively low level of harm for animals in contrast to crushing models, which are invasive and associated with the risk of infection. This explains why CTX-induced injury models have been widely used in exploring the mechanisms of muscle regeneration. To understand the regeneration mechanisms in CTX-induced injury models, we explored all the studies and summarized the characteristics and injury positions, different models of CTX injury, and functional factors involved in the process of regeneration. The results show that the process of regeneration is similar in different mouse strains but that differences exist between gender. Regeneration is impaired in obese, diabetic, and aging mice, whereas exercise, electrical stimulation, and overloading facilitate the regeneration of damaged muscle. Non-SCs transplanted in damaged muscle following CTX injury can also differentiate into myogenic cells and facilitate myogenesis. The emphasis throughout was on the process of regeneration, the changes in the functional proteins involved in the processes of clearance of necrotic fiber debris, M1 to M2 macrophage conversion, SC activation, myoblast proliferation, differentiation and fusion, and fibrosis and calcification, which influence the final outcome of the regenerative activity. However, the inflammatory process in muscle injury and repair is complex, with different effects on muscle regeneration observed in various studies. Additionally, angiopoiesis and neurogenesis also influence the outcome of regeneration, which are easily ignored. Thus, further experiments are needed to explore the mechanisms of inflammatory response during muscle regeneration.

## Figures and Tables

**Table 1:** Different mice models on CTX-induced regeneration in skeletal muscle.

Author, Year	Injury Portions	Mouse Models	Targets	Regeneration (Impair/Improve)	Ref.
Mouisel E, 2010	Right tibialis anterior	5 weeks,5, 12 and 18–24 months mdx mice	Different ages	Impair	[28]
Fearing CM, 2016	Right hind limb anterior and post compartments	Male and female C57BL/6J mice; young:4-6 months, middle:12–19 months, old 25–30 months and very old:32–33 months	Ages and sex	Impair; ——	[40]
Takahashi Y, 2021	Left tibialis anterior	22 weeks C57BL/6-WT, C57BL/6-Akita, KK/Ta-WT, and KK/Ta-Akita male mice	Diabetes	Impair	[20]
Vignaud A, 2007	Right tibialis anterior	3–4 months STZ-treated Swiss and Akita male mice	Diabetes	Impair	[21]
Chaiyasing R, 2021	Right tibialis anterior	12 weeks ovariectomized and normal C5BL/6JJcl female mice	Estrogen	Impair	[41]
Rebalka IA, 2017	Tibialis anterior	10–12W STZ-treated and normal male C57BL/6J mice	Fluvastatin	Impair	[42]
Nguyen MH, 2011	Extensor digitorum longus	14–16 weeks leptin-deficient, leptin receptor-mutant mice and a group of C57BL/6 mice fed a high-fat diet	ob/ob and db/db	Impair	[22]
D’Souza DM, 2015	Left gastrocnemius-plantaris, tibialis anterior, quadriceps	16 weeks male C57BL/6J mice fed a high-fat diet	Diet-induced obesity	Impair	[24]
Jinno N, 2014	Right gastrocnemius	8 weeks male C57BL/6 mice: a marginally zinc-deficient diet-fed group, a zinc-adequate diet-fed group and a zinc-high diet-fed group	Zinc	Impair	[36]
Matsuba Y, 2009	Soleus muscle	gravitational unloading	Gravitational unloading	Impair	[32]
Jeong J, 2013	Tibialis anterior and gastrocnemius	2–4 months C57BL/6 male mice treated with STZ, 20–24 months C57BL/6 male mice and C57BL/6J-Ins2Akita mice	Diabetes	Impair	[43]
Inaba S, 2018	Tibialis anterior	cancer cachexia	Cancer cachexia	Impair	[26]
McHale MJ, 2012	Right hind limb anterior and posterior compartment	4–6 months ovariectomized, castrated and normal male and female C57BL/6J mice	Sex hormones	——	[44]
Patsalos A, 2017	Tibialis anterior	2–6 months BoyJ, C57BL/6J male mice with or without irradiation and Pax7 Cre-Rosa26 DTA mice	Irradiation	Impair	[29]
Ikeda Y, 2019	Gastrocnemius	8 weeks male C57BL/6J mice with or without iron overload	Iron	Impair	[45]
Attia M, 2017	Right tibialis anterior	10 weeks myasthenia gravis and normal female C57BL/6J mice	Myasthenia gravis	Impair	[46]
Saliu TP, 2022	Left tibialis anterior and gastrocnemius	9 weeks normal and NAFLD male CD-1 mice	NAFLD	Impair	[47]
Rahman FA, 2020	Left tibialis anterior	Young (3 months) and old (18 months) male C57BL/6J mice	Aging	Impair	[48]
Kohno S, 2012	Soleus	C57BL/6J mice with or without tail suspension	Unloading	Impair	[38]
Kimoloi S, 2022	Tibialis anterior	Both male and female K320E^skm^ and K320E^msc^ transgenic mice	Mitochondrial DNA alterations	Impair	[39]
Paiva-Oliveira EL, 2017	Right gastrocnemius	Isogenic 6–8 weeks C3H/HeJ (TLR4 defective), C3H/HeN(TLR4 WT), C57BL/6 (WT) and TLR-4 knockout male mice	Different strains	——	[49]
Yoshioka K, 2021	Tibialis anterior and masseter	C57BL/6J and mdx mice	Position specificity	——	[50]
Joanisse S, 2016	Tibialis anterior	24 months old male C57BL/6J mice with or without exercise and 8 weeks young male mice	Exercise and age	Improve; impair	[33]
Nagata K, 2013	Tibialis anterior	8 weeks C57BL/6J mice with ultrasound exposure	Ultrasound	Improve	[51]
Morioka S, 2008	Soleus	10 weeks male C57BL/6J mice with or without functional overloading	Functional overloading	Improve	[37]
Fujiya H, 2015	Left tibialis anterior	7 weeks male C57BL/6J mice with or without microcurrent electrical neuromuscular stimulation (MENS)	MENS	Improve	[35]

**Table 2:** The mechanism of inflammatory infiltration, conversion of macrophages, and elimination of the necrotic debris.

Author, Year	Injury Portions	Target Molecule/ Drug	Target Process	Expression	Effects (Positive/Negative)	Regeneration (Impair/Improve)	Ref.
Shi D, 2018	Tibialis anterior	CaMKIV	Infiltration of macrophages	Up/Down	Positive/Negative	Impair/improve	[57]
Neves Jde C, 2015	Right tibialis anterior	Neuraminidase-1	Inflammatory response; myofiber maturation	Down	Positive; Negative	Impair	[58]
Liao ZH, 2019	Tibialis anterior	Estrogen signaling	Inflammation infiltration; conversion of macrophages from M1 to M2	Down	Positive; Negative	Impair	[59]
Kohno S, 2011	Tibialis anterior	Cbl-b	Cytotoxic T-cell infiltration	Down	Positive	Impair	[60]
Wang H, 2014	Right tibialis anterior and hindlimb posterior compartment	Monocyte/ macrophage	Monocyte and macrophages recruitment; conversion of macrophages from M1 to M2	——	Positive; Negative	Impair	[61]
Park CY, 2010	Gastrocnemius and soleus	skNAC	Inflammation infiltration and myonecrosis	Down	Positive	Impair	[62]
Shi H, 2010	Right tibialis anterior	MKP-1	Inflammation; myoblast proliferation; differentiation	Down	Positive; Negative; Positive	Impair	[63]
Hu J, 2019	Tibialis anterior	CaM signaling	Inflammatory response	Up	Positive	Improve	[64]
Manoharan P, 2019	Gastrocnemius	Klf2	Inflammatory response	Down	Positive	Improve	[65]
Gao Y, 2012	Unilateral tibialis anterior	STAT1	Inflammatory response	Down	Positive	Improve	[66]
Kozakowska M, 2018	Gastrocnemius	Hmox1	Inflammation and SC proliferation	Down	Positive	Improve	[67]
Koh, 2005	Extensor digitorum longus	PAI-1	Macrophage and SC migration	Down	Positive	Improve	[2]
Zhang, 2020	Tibialis anterior and gastrocnemius	IFN-γ/CXCL10/ CXCR3	Macrophages and myoblast proliferation	——	Positive	Improve	[68]
Yaden BC, 2014	Right Gastrocnemius	Activin A	Macrophage infiltration	Up	Positive	Improve	[69]
Mothe-Satney I, 2017	Left Tibialis Anterior	PPARβ	Macrophage recruitment	Up	Positive	Improve	[70]
Tanaka Y, 2019	Tibialis Anterior	APN	Elimination of the necrotic fibers	Up	Positive	Improve	[71]
Dinulovic I, 2016	Tibialis Anterior	PGC-1α	Conversion of macrophages from M1 to M2	Up/Down	Positive; Negative	Improve/Impair	[56]
Sugihara H, 2018	Tibialis Anterior	PGRN	Prolonged Persistence of M2 Macrophages	Down	Positive	Improve	[72]
Lo Sicco, 2017	Tibialis Anterior	Extracellular vesicles released by human adipose derived-MSCs	Conversion of macrophages from M1 to M2	Up	Positive	Improve	[73]
Yang M, 2022	tibialis anterior	Balenine	Phagocytosis ability of macrophages	——	Positive	improve	[74]
Cardoso ES, 2016	Gastrocnemius	Thymol	Inflammatory response	——	Negative	Improve	[75]
Wang ZG, 2021	Gastrocnemius	Conversion of n-6 to n-3 PUFAs	Inflammatory response; SC activation	Up	Negative; Positive	Improve	[76]
Chaweewannakorn C, 2018	Unilaterally tibialis anterior	IL-1a/β	Inflammatory response	Down	Negative	Impair	[77]
Senf SM, 2013	Tibialis anterior	Hsp70	Inflammatory response	Down	Negative	Impair	[78]
Mojumdar K, 2016	Tibialis anterior	TLR2	Macrophage accumulation; elimination of the necrotic fibers	Down	Negative; Negative	Impair	[79]
Varga T, 2013	Tibialis anterior	NUR77	Macrophage development	Down	——	Impair	[80]
Ochoa O, 2007	Right anterior and posterior compartment	CCR2	Macrophage recruitment and angiogenesis and VEGF production	Down	Negative	Impair	[81]
Zhang C, 2013	Tibialis anterior and gastrocnemius	IL-6/STAT3	Infiltration of macrophages and myoblast proliferation	Down	Negative	Impair	[82]
Zhang J, 2014	Tibialis anterior	CD8	Macrophage recruitment	Down	Negative	Impair	[83]
Martinez CO, 2010	Right tibialis anterior and hindlimb posterior compartment	CCR-2/MCP-1	Macrophage recruitment	Down	Negative	Impair	[84]
Krause MP, 2013	Left tibialis anterior and gastrocnemius-plantaris-soleus	Diabetes	Macrophages infiltration	——	——	Impair	[23]
Cheng M, 2008	Extensor digitorum longus and tibialis anterior	IFN-γ	Macrophages infiltration; myoblast proliferation	Down	Negative	Impair	[85]
Zhang, 2017	Tibialis anterior and gastrocnemius	C3a	Monocyte/macrophage infiltration	Down	Negative	Impair	[86]
Sun D, 2009	Right hind limb anterior and posterior compartment	CCR2	Recruitment of macrophages and neutrophils	Down	Negative	Impair	[87]
Nishimura D, 2015	Tibialis anterior	ADAM8	Elimination of the necrotic fibers	Down	Negative	Impair	[88]
AI-Zaeed N, 2021	Tibialis anterior	TAM kinase receptor Mer	Elimination of the necrotic fibers and conversion of macrophage from M1 to m2s	Down	Negative	Impair	[89]
Zhang J, 2019	Tibialis anterior	SRB1	Elimination of the necrotic fibers and conversion of macrophage from M1 to M2	Down	Negative	Impair	[90]
Jin R M, 2018	One or more hindlimb muscles	Preexisting inflammatory environment	Conversion of macrophage from M1 to M2	——	Negative	Impair	[91]
Bronisz-Budzyńska I, 2020	Gastrocnemius	Nrf2	Inflammatory response	Down	Positive	No effects	[92]
Tarban N, 2022	Tibialis anterior	Retinol saturase	Phagocytosis ability of macrophages	Down	Negative	No effects	[93]
Dalle S, 2020	Tibialis anterior	Ibuprofen	Inflammatory response	——	Negative	No effects	[94]
Shen W, 2008	Gastrocnemius	Macrophage, TFG-β1 and COX-2	Inflammatory response	——	——	——	[95]
Rousseau AS, 2021	Left tibialis anterior	PPARβ/δ	T cell dynamic	Down	——	——	[96]

**Table 3:** The mechanism of SC activation, myoblast proliferation, differentiation, and fusion.

Author, Year	Injury Portions	Target Molecule/ Drug	Target Process	Expression	Effects (Positive/Negative)	Regeneration (Impair/Improve)	Ref.
Schaaf G J, 2018	Quadriceps femoris and gastrocnemius	Acid alpha glucosidase	SC activation	Down	Negative	Impair	[115]
Serra C, 2013	Left tibialis anterior	Testosterone	SC activation	Up	Positive	Improve	[116]
Liu Q, 2021	Left tibialis anterior	Salvador	SC activation and angiogenesis	Down	Positive	Improve	[117]
Zeng L, 2010	Tibialis anterior or gastrocnemius	Insl6	SC activation and proliferation	Up/Down	Positive/Negative	Improve/impair	[108]
Shelar SB, 2016	Tibialis anterior	Nrf2	SC activation and proliferation	Down	Negative	Impair	[113]
Rebalka IA, 2018	Left tibialis anterior or left gastrocnemius	Lcn2	SC activation and fibrosis	Down	Negative	Impair	[114]
Zeng P, 2016	Right tibialis anterior	Mir-378/IGF1R	SC activation and differentiation	Up	Negative	Impair	[118]
Nissar AA, 2011	Tibialis anterior	Xin	SC activation	Down	Negative	Impair	[111]
Lagalice L, 2018	Tibialis anterior	Acid alpha glucosidase	SC activation	Down	Negative	Impair	[119]
Mizbani A, 2016	Tibialis anterior	Mirna-501	SC activation	Down	Negative	Impair	[120]
Fiore PF, 2020	Tibialis anterior	Pkcθ	SC self-renewal	Down	Positive	Improve	[121]
Fortier M, 2013	Tibialis anterior	S1pr3	SC proliferation	Down	Positive	Improve	[122]
Cai S, 2020	Tibialis anterior	Mll1/myf5	SC proliferation	Up	Positive	Improve	[123]
Sincennes MC, 2021	Tibialis anterior	Pax7 acetylation	SC pool	Down	Positive	Improve	[124]
Naito T, 2009	Tibialis anterior	G-csf	SC number	Up	Positive	Improve	[125]
Ohno Y, 2016	Left soleus	Mstn	SC number	Down	Positive	Improve	[31]
Price FD, 2014	Tibialis anterior	Jak/stat	SC number	Down	Positive	Improve	[126]
Hillege MMG, 2022	Tibialis anterior	TGF-β signaling	SC number	Down	Positive	Improve	[112]
Angione AR, 2011	Tibialis anterior and gastrocnemius	Pparδ	SC number and proliferation	Down	Negative	Impair	[127]
Nishizawa S, 2013	Left soleus	Hsf1	SC number and proinflammatory response	Down	Negative	Impair	[128]
Ahrens HE, 2018	Right tibialis anterior	Klotho	SC number and function	Down	Negative	Impair	[129]
Sakamoto K, 2019	Forearm muscle	R3hdml	SC number	down	Negative	Impair	[130]
Bye-A-Jee H, 2018	Tibialis anterior	ZFP36L1 and ZFP36L2	SC number	Down	Negative	Impair	[131]
Tonami K, 2013	Left tibialis anterior	Capn6	Myoblast differentiation	Down	Positive	Improve	[132]
Accornero F, 2014	Tibialis anterior	TGF-β	SC number and activity; decreased degeneration	Down	Positive; Positive	Improve	[133]
Van Ry PM, 2014	Tibialis anterior	Laminin-111	SC pool; fibrosis	Up	Positive; Negative	Improve	[134]
Hosoyama T, 2011	Tibialis anterior	Rb1	SC pool; differentiation	Down	Positive; Negative	Impair	[135]
Rion N, 2019	Unilaterally tibialis anterior	mTORC2	SC pool replenishment	Down	Negative	Impair	[136]
Yoshida T, 2013	Unilateral gastrocnemius	Angiotensin II	SC pool and proliferation	Up	Negative	Impair	[137]
Armand AS, 2011	Soleus or EDL	AIF	SC pool	Down	Negative	Impair	[138]
Milanesi A, 2017	Right tibialis anterior or quadriceps femoris	Thyroid hormone receptor alpha	SC pool	Down	Negative	Impair	[139]
Castets P, 2011	Unilateral tibialis anterior and soleus muscles	SelN	SC pool	Down	Negative	Impair	[140]
Fujimaki S, 2018	Right tibialis anterior	Notch1/Notch2	SC pool and proliferation; myoblast differentiation; fibrosis	Down	Negative; Positive; Positive	Impair	[141]
Johnston AP, 2011	Tibialis anterior	Ang II	SC number; myoblast differentiation	Down	Positive; Negative	Impair	[142]
Jaafar M N, 2016	Right tibialis anterior	Primary cilium	SC self-renewal	——	——	——	[143]
Leung C, 2020	Tibialis anterior/Extensor digitorum longus	Lgr5	SC replenish and myofiber formation	——	Positive	Improve	[144]
Buono R, 2012	Tibialis anterior and quadriceps	NO signaling	SC self-renewal and proliferation	Down	Negative	Impair	[145]
Urciuolo A, 2014	Tibialis anterior	Collagen VI	SC self-renewal	Down	Negative	Impair	[146]
Alexeev V, 2014	Left gastrocnemius muscle	Adipose-derived stem cells	Migration of SCs	——	Positive	Improve	[147]
Brien P, 2013	Tibialis anterior/extensor digitorum longus muscle group	P38α	Myoblast proliferation; differentiation	Down	Positive; Negative	Impair	[148]
Hawke TJ, 2003	Tibialis anterior	p21	Myoblast proliferation; differentiation	Down	Positive; Negative	Impair	[149]
Cortez-Toledo O, 2017	Tibialis anterior	Nur77	Myoblast proliferation	Down	Negative	No effects	[150]
Alves, 2019	Rectus femoral muscle	Kinin-B2 receptor	Myoblast proliferation; differentiation	Down	Positive; Negative	Impair	[4]
Wu, 2014	Right tibialis anterior	Duxbl	Myoblast proliferation and differentiation	Up	Positive; Negative	Impair	[151]
Chen Y, 2012	Tibialis anterior	miR-351	Myoblast proliferation and differentiation	Up/Down	Positive/Negative	Improve/impair	[152]
Tseng C, 2019	Gastrocnemius	Sod1/Cat	Myoblast proliferation and differentiation	Up	Positive	Improve	[153]
Jia Y, 2012	Gastrocnemius	EPO	Proliferation and survival of the SCs	——	Positive	Improve	[154]
Shibasaki H, 2019	Tibialis anterior	miR-188	Myoblast fusion	Up/Down	Positive/Negative	Improve/impair	[155]
Hawke TJ, 2007	Tibialis anterior	Xin	Myoblast proliferation and migration	Down	Positive	Improve	[109]
Meng, 2014	Tibialis anterior	RNF13	Myoblast proliferation and differentiation	Up	Positive	Improve	[104]
Lee EJ, 2021	Left gastrocnemius	Glycyrrhiza uralensis-extracted compounds	Myoblast proliferation and differentiation	——	Positive	Improve	[156]
Galimov A, 2016	Tibialis anterior	FGF2	Myoblast proliferation	Up	Positive	Improve	[107]
Armand, 2003	Soleus	FGF6	Myoblast proliferation	Down/Up	Negative/Positive	Impair/improve	[157]
Shi, 2010	Tibialis anterior and gastrocnemius	Tceal7	Myoblast proliferation; differentiation	Up	Negative; Positive	——	[1]
Pessemesse L, 2019	Right tibialis anterior	p43	Myoblast proliferation	Down/Up	Negative/Positive	Impair/improve	[158]
Ye, 2016	Gastrocnemius	CaMKK2	Myoblast proliferation and differentiation	Up/Down	Negative/Positive	Impair/ Improve	[159]
Minetti GC, 2014	Tibialis anterior	Gαi2	Myoblast proliferation, differentiation and fusion	Down	Negative	Impair	[160]
Zhang CC, 2021	Tibialis anterior	Cyp4a14	Myoblast proliferation and differentiation and inflammatory response	Down	Negative	Impair	[161]
Ding, 2021	Tibialis anterior	Tfr1	Myoblast proliferation and differentiation	Down	Negative	Impair	[162]
Naito M, 2016	Tibialis anterior	Dnmt3a	Myoblast proliferation	Down	Negative	Impair	[163]
Bae, 2020	Tibialis anterior	Cdon	Myoblast proliferation and senescence	Down	Negative	Impair	[164]
Rooney JE, 2009	Left tibialis anterior	α7 integrin	Myoblast proliferation and differentiation	Down	Negative	Impair	[165]
Katsushi, 2020	Tibialis anterior	Bach 1	Myoblast proliferation and differentiation	Down	Negative	Impair	[166]
Yamashita, 2016	Gastrocnemius	FOXO1	Myoblast proliferation	Up	Negative	Impair	[167]
Al-Sajee D, 2015	Left tibialis anterior, gastrocnemius/ Plantaris/soleus, quadriceps muscles	Xin	Myoblast proliferation	Down	Negative	Impair	[110]
Girgenrath, 2006	Tibialis anterior	Fn14	Myoblast proliferation	Down	Negative	Impair	[168]
Martinet C, 2016	Tibialis anterior	H19	Myoblast proliferation	Down	Negative	Impair	[169]
Yahiaoui, 2008	Tibialis anterior	MCP-1	Myoblast proliferation	Up	Negative	Impair	[170]
Kursaka, 2017	One leg of tibialis anterior	Egr3	Myoblast proliferation	Down	Negative	Impair	[171]
Ochiai N, 2016	Tibialis anterior	fad24	Myoblast proliferation	Down	Negative	Impair	[172]
Zanou, 2012	Tibialis anterior and extensor digitorium longus	Trpc1	Myoblast migration and differentiation	Down	Negative	Impair	[173]
Yablonka-Reuveni Z, 2015	Unilateral tibialis anterior	FGFR1	Myoblast proliferation	Down	Negative	No effects	[174]
Ohtsubo, 2017	Gastrocnemius	APOBEC2	Myoblast differentiation and fusion	Down	Positive	Improve	[8]
He, 2019	Extensor digitorum longus	Nicotine	Myoblast differentiation	——	Positive	Improve	[175]
Wu, 2007	Tibialis anterior	Sema4C	Myoblast differentiation	Up/Down	Positive/Negative	Improve/impair	[176]
Liu, 2012	Tibialis anterior	miR-206	Myoblast differentiation	Up	Positive	Improve	[177]
Song, 2018	Tibialis anterior	Linc-smad7	Myoblast differentiation	Up	Positive	Improve	[178]
Gatta L, 2017	Right tibialis anterior	Trimetazidine	Myoblast differentiation	——	Positive	Improve	[179]
Gagan, 2011	Tibialis anterior	miR-378	Myoblast differentiation	Up	positive	Improve	[180]
Mikami T, 2012	Tibialis anterior	Chondroitin sulfate	Myoblast differentiation	Down	Positive	Improve	[181]
Lee KP, 2015	Hindlimb muscle	miR-431	Myoblast differentiation	Up	positive	Improve	[27]
Storbeck CJ, 2013	Tibialis anterior	SLK	Myoblast differentiation	Down	Positive	Improve	[182]
Lei, 2013	One leg of tibialis anterior	MMP-13	Myoblast migration	Down/Up	Negative/Positive	Impair/improve	[183]
Das, 2017	Tibialis anterior	ACL	Myoblast differentiation	Down/Up	Negative/Positive	Impair/improve	[184]
Yoshida T, 2014	Gastrocnemius	AT2R	Myoblast differentiation	Down/Up	Negative/Positive	Impair/improve	[185]
Huang Y, 2016	Right tibialis anterior	Ccndbp1	Myoblast differentiation	Down/Up	Negative/Positive	Impair/ Improve	[186]
Chen SE, 2007	Soleus	TNF-α	Myoblast differentiation	Down/Up	Negative/Positive	Impair/ Improve	[187]
He, 2021	Tibialis anterior	IRE1a	Myoblast differentiation and hypertrophy	Down/Up	Negative/ Positive	Impair/improve	[188]
Luca E, 2020	Tibialis anterior	miRNA network	Myoblast differentiation	Down	Negative	Improve	[189]
Esteca MV, 2020	Left tibialis anterior	Parkin	Myoblast differentiation	Down	Negative	Impair	[190]
Ishii A, 2001	Tibialis anterior	Tensin	Myoblast differentiation and fusion	Down	Negative	Impair	[191]
Zhang M, 2020	Tibialis anterior of one limb	Rbm24	Myoblast differentiation	Down	Negative	Impair	[106]
Lee, 2015	Tibialis anterior	miR-431	Myoblast differentiation	Down	Negative	Impair	[192]
Fan, 2018	Tibialis anterior	Hsp70	Myoblast differentiation	Down	Negative	Impair	[16]
Cerquone, 2018	Tibialis anterior	PAK1	Myoblast differentiation	Down	Negative	Impair	[193]
Lee, 2020	Tibialis anterior	PHF20	Myoblast differentiation	Up	Negative	Impair	[194]
Hayashi, 2016	Tibialis anterior	Klf5	Myoblast differentiation	Down	Negative	Impair	[3]
Lee, 2015	Tibialis anterior	AKAP6	Myoblast differentiation	Down	Negative	Impair	[195]
Li, 2020	Tibialis anterior	LRTM1	Myoblast differentiation	Down	Negative	Impair	[196]
Lin, 2019	Left gastrocnemius	MPM	Myoblast differentiation	Down	Negative	Impair	[197]
Harada, 2018	Tibialis anterior	H3mm7	Myoblast differentiation	Down	Negative	Impair	[198]
Tetsuaki, 2009	Tibialis anterior	CT-1	Myoblast differentiation	Up	Negative	Impair	[199]
Paul, 2012	Tibialis anterior	COPR5	Myoblast differentiation	Down	Negative	Impair	[200]
Faralli, 2011	Tibialis anterior and gastrocnemius of one hind limb	Tshz3	Myoblast differentiation	Up	Negative	Impair	[201]
Liu N, 2014	Tibialis anterior	MEF2A, C and D	Myoblast differentiation	Down	Negative	Impair	[202]
Kielbasa OM, 2011	Unilateral tibialis anterior	Myospryn	Myoblast differentiation	Up	Negative	Impair	[203]
Verpoorten S, 2020	Tibialis anterior	CD36	Myoblast differentiation	Down	Negative	Impair	[204]
Andrée B, 2002	Right gastrocnemius and soleus	Pop	Myoblast differentiation	Down	Negative	Impair	[205]
Paolini A, 2018	Tibialis anterior	Autophagy	Myoblast differentiation	Down	Negative	Impair	[206]
Clow C, 2010	Tibialis anterior	BDNF	Myoblast differentiation	Down	Negative	Impair	[207]
Marshall JL, 2012	Left quadriceps	SSPN	Myoblast differentiation	Down	Negative	Impair	[208]
Chen SE, 2005	Soleus	TNF-α	Myoblast differentiation	Down	Negative	Impair	[209]
Ravel, 2014	Tibialis anterior	Staufen1	Myoblast differentiation	Up	Negative	Impair	[210]
Langsdorf A, 2007	Tibialis anterior	Sulfs	Myoblast differentiation	Down	Negative	Impair	[211]
Liu H, 2011	Tibialis anterior and soleus	β3-Integrin	Myoblast differentiation	Down	Negative	Impair	[212]
Watanabe S, 2007	Right tibialis anterior	Lbx1	Myoblast differentiation	Down	Negative	Impair	[213]
Fu D, 2015	Tibialis anterior	Mdm2	Myoblast differentiation	Down	Negative	Impair	[214]
Schroer, 2019	Tibialis anterior	RGS12	A switch from myoblast proliferation to differentiation	Down	Negative	Impair	[215]
Lim S, 2013	Left tibialis anterior	CBR1	Myoblast differentiation	Down	Negative	——	[216]
Mammen AL, 2009	Right tibialis anterior	Mi-2	Myoblast differentiation	Up	Negative	——	[217]
Wu Z, 2020	Tibialis anterior	Andrographolide	Myoblast differentiation and fusion	——	——	Improve	[218]
Kurosaka, 2021	Left tibialis anterior	STAT6	Myoblast differentiation and fusion	Down	——	Improve	[219]
Budai Z, 2021	Tibialis anterior	TG2	Myoblast fusion and conversion of macrophages from M1 to M2	Down	Negative	Impair	[102]
Vijayakumar A, 2013	Unilateral tibialis anterior	IGF-1R	Myoblast fusion	Down	Negative	Impair	[220]
Pryce BR, 2017	Unilateral tibialis anterior	SLK	Myoblast fusion	Down	Negative	Impair	[221]
Redelsperger, 2016	One tibialis anterior	Syncytin	Myoblast fusion	Down	Negative	Impair	[222]
Kaspar, 2013	Tibialis anterior	3′ untranslated region of c-Myb	Myoblast fusion	Down	Negative	Impair	[223]
Griffin, 2016	Left tibialis anterior and gastrocnemius	ANO5	Myoblast fusion	Down	Negative	Impair	[224]
Trapani L, 2012	Right tibialis anterior	HMGR	Myoblast fusion	Down	Negative	Impair	[225]
Hamoud, 2018	Tibialis anterior	BAI3	Myoblast fusion	Down	Negative	Impair	[226]
Tamilarasan K P, 2012	Gastrocnemius	Lipid accumulation	Myoblast fusion	Up	Negative	Impair	[227]
Yoshida N, 2019	Tibialis anterior	(P)RR	Myoblast fusion	Up	Negative	Impair	[228]
Shibasaki H, 2019	Tibialis anterior	miR-188	Myoblast fusion	Up/Down	Positive/Negative	Improve/impair	[155]
Youm TH, 2019	tibialis anterior	Nox4/ROS	Myoblast fusion	——	Positive	Improve	[229]
Teng, 2015	One tibialis anterior	PLD1	Myoblast fusion	Up	Positive	Improve	[230]
Krause MP, 2013	Left tibialis anterior	Mustn1	Myoblast fusion	Up	Positive	Improve	[231]
Kurosaka, 2016	Tibialis anterior	TRPV I	Myoblast fusion	Up	Positive	Improve	[232]
Singhal N, 2015	Gastrocnemius, quadriceps, tibialis anterior	Galgt1	Myoblast fusion; SC apoptosis	Down	Negative; Positive	Impair	[233]
Yalvac ME, 2017	Left tibialis anterior and left gastrocnemius	Calpain-3	Myoblast fusion; fibrosis	Down	Negative; Positive	Impair	[234]
Ogawa, 2015	Tibialis anterior	Dcx	Myofiber maturation	Down	Negative	Impair	[235]
Ohno Y, 2019	Right tibialis anterior	Lactate	Myotube formation	Up	Positive	Improve	[236]
Hoshino S, 2013	Right tibialis anterior	CHC22	Myofiber maturation	Up	Negative	Impair	[237]
Hu Z, 2010	Unilateral tibialis anterior	PTEN	Myofiber maturation; fibrosis	Down	Positive	Improve	[25]
Agbulut O, 2001	Gastrocnemius and soleus or tibialis anterior	Desmin	Myofiber maturation; neuromuscular junctions	Down	Negative	Impair	[238]
Cicchillitti, 2012	Tibialis anterior	miR-210	Myoblast differentiation	Down	——	No effects	[239]
Piccioni A, 2014	Tibialis anterior	Shh	Activated SCs	Up	——	Improve	[240]
Ceco E, 2021	Left tibialis anterior	Elevated CO_2_ exposure	Myoblast differentiation and fusion	——	——	Impair	[30]
Liu, 2017	Tibialis anterior	Twist2	Maintain SC state	——	——	——	[241]

**Table 4:** Fibrosis, calcification, and apoptosis.

Author, Year	Injury Portions	Target Molecule/ Drug	Target Process	Expression	Effects (Positive/Negative)	Regeneration (Impair/Improve)	Ref.
Heredia JE, 2013	Unilateral tibialis anterior	IL-4	FAP proliferation	Down	Negative	Impair	[55]
Vumbaca S, 2021	Tibialis anterior, quadriceps, and gastrocnemius	IL1a/IL1β and extracellular vesicles	FAP proliferation and differentiation	Up	Positive	——	[248]
Zhao L, 2020	Tibialis anterior	RA signaling	FAP proliferation	Up	positive	Improve	[246]
Zanotti S, 2018	Tibialis anterior	Exosome miR-199a-5p/CAV1	Fibrosis	Up	Positive	Impair	[247]
Horii N, 2018	Tibialis anterior	C1q/Wnt and resistance training	Fibrosis	Up	positive	Impair	[34]
Murray, 2017	Tibialis anterior	αV intergin	Fibrosis	Down	Negative	Improve	[245]
Burks, 2011	Tibialis anterior	Losartam	Fibrosis	——	Negative	Improve	[250]
Stepien DM, 2020	Left tibialis anterior	TGF-β1	Fibrosis	Down	Negative	Improve	[249]
Bosnakovski D, 2022	Tibialis anterior	a prior DUX4 burst	Fibrosis	——	Positive	——	[251]
Ding, 2016	Tibialis anterior	TAR RNA-binding protein (Trbp)	Fibrosis; myofiber formation	Down	Positive; Negative	Impair	[244]
Ogasawara S, 2018	Left gastrocnemius	CatK	Fibrosis; inflammation and cell apoptosis	Down	Negative	Improve	[252]
Lee SJ, 2010	Gastrocnemius	Follistatin	Fibrosis; myofiber maturation	Down	Negative	Impair	[253]
Rinaldi F, 2016	Tibialis anterior	GDF11	Collagen deposition	Up	Positive	No effects	[254]
Mignemi NA, 2017	Posterior compartments of the lower extremities	Plasmin	Calcification	Down	Negative	Impair	[255]
Lee YS, 2013	Right gastrocnemius	Gasp1 and/or Gasp2	Calcified fibers and fibrosis	Down	Positive	Impair	[256]
Zhao Y, 2009	Tibialis anterior	——	Dystrophic calcification	——	——	——	[257]
Lounev V Y, 2009	Quadriceps	Tie2-expressing endothelial precursors	Heterotopic ossification	——	——	——	[258]
Drouin G, 2019	——	Hypoxic state	Heterotopic ossification	——	——	——	[259]
Arsic N, 2004	Tibialis anterior	VEGF	Apoptosis	Up	Negative	improve	[260]
Sinha-Hikim I, 2007	Gastrocnemius	JNK and iNOS signaling	Cell apoptosis	Down	Negative	improve	[261]
Min K, 2017	Gastrocnemius/ Soleus	MKP-5	myofiber apoptosis	Down	Negative	improve	[262]
Tjondrokoesoemo A, 2016	Tibialis anterior	serpina3n	Stabilization of myofiber plasm membrane	Up	Positive	Improve	[263]

**Table 5:** Angiogenesis and neurogenesis in CTX-induced injury models.

Author, Year	Injury Portions	Target Molecule/ Drug	Target Process	Expression	Effects (Positive/Negative)	Regeneration (Impair/Improve)	Ref.
Bellamy LM, 2010	Unilateral tibialis anterior	Angiotensin II	Angiogenesis	Down	Negative	Impair	[266]
Ieronimakis N, 2012	Tibialis anterior, quadriceps, gastrocnemius	Bone marrow-derived cells	Angiogenesis	——	Positive	Improve	[265]
Mellows B, 2017	Tibialis anterior	Extracellular vesicles-derived from amniotic fluid stem cell	Angiogenesis	——	Positive	Improve	[267]
Hosaka Y, 2002	Right tibialis anterior	α1-Syntrophin	Hypertrophy and neuromuscular junctions	Down	Positive; Negative	Improve	[268]
Daneshvar N, 2020	Left tibialis anterior	Premature satellite cell activation	Maturation of neuromuscular junctions	Up	Negative	Improve	[269]
Kurosaka M, 2021	Tibialis anterior	M2 macrophage	Motor innervation regeneration	——	——	Improve	[219]
Sawano S, 2014	Tibialis anterior	M2 macrophage	Motor innervation regeneration	——	——	Improve	[270]
Randazzo D, 2019	Unilaterally tibialis anterior	Tubb6	Microtubule organization	Up	Negative	——	[15]

**Table 6:** Single gene in the CTX-induced skeletal muscle injury model.

Author, Year	Injury Portions	Target Molecule	Expression	Regeneration (Impair/Improve)	Ref.
Kim DS, 2015	Left tibialis anterior	TLR2	Down	Improve	[272]
Oikawa S, 2019	Tibialis anterior	Dicer	Down	Impair	[273]
Hiramuki Y, 2015	Tibialis anterior	Mest	Down	Impair	[274]
Norton CR, 2013	Tibialis anterior	Snai1/Snai3	Down	No effects	[275]
Call JA, 2017	Left tibialis anterior and left flexor digitorum longus	Ulk1	Down	——	[276]
Chaturvedi N, 2020	Left gastrocnemius	S100A1	Down	——	[277]
Parks CA, 2019	Left tibialis anterior	Trim33	Down	No effects	[278]
Goetsch SC, 2005	Gastrocnemius	Filamin C	Up	——	[279]
Wardrop KE, 2011	Left tibialis anterior	LYVE-1	Down	——	[280]
Merkulova T, 2000	Extensor digitorum longus and tibialis anterior	β enolase	Up	——	[281]
Yuasa K, 2008	Tibialis anterior	miR-206	Up	——	[282]
Casciola-Rosen L, 2012	Right anterior tibilias	Aldolase A	Up	——	[283]
Mammen AL, 2011	Right tibialis anterior	UFD2a	Up	——	[284]
Nakamura K, 2010	Right gastrocnemius	GNE	Up	——	[285]
Sato Y, 2013	Left gastrocnemius	Sema3A	Up	——	[286]
Garry, 2000	Hind limbs	MNF	Down	Impair	[287]
Kemp MW, 2009	Tibialis anterior	Syncoilin	Up	——	[288]
Miura P, 2005	Right tibialis anterior	Utrophin A	Up	——	[289]
Wang Q, 2022	Tibialis anterior	Tsukushi	Down	Impair	[290]
McCullagh KJ, 2008	Unilateral tibialis anterior	Syncoilin	Down	No effects	[291]
Demonbreun AR, 2010	Quadriceps or gastrocnemius/soleus	Ferlin	Up	——	[292]
Maeda Y, 2017	Right tibialis anterior	CXCL12	Up	Improve	[293]
Darabi R, 2008	Tibialis anterior	Pax3	Up	Improve	[294]
Di Rocco A, 2015	Tibialis anterior	RARγ	Down	Impair	[295]
Bryer SC, 2007	Extensor digitorum longus and tibialis anterior	uPAR	——	No effects	[296]
Bryan BA, 2005	Tibialis anterior	GEFT	Up	Improve	[297]
Mathes AL, 2011	Tibialis anterior	TLR-3	Down	Impair	[298]
Wu G, 2010	Forelimb leg muscle	Chkb	Down	No effects	[299]
Yan Z, 2003	Tibialis anterior	E2f1	Down	Impair	[5]
Fujita R, 2014	Left tibialis anterior	IL-6R	Down	Improve	[300]
Wu G, 2009	Gastrocnemius	Chkb	Down	Impair	[301]
Wada E, 2019	Tibialis anterior	Emerin and lamin A/C	Down	Impair	[302]
Mofarrahi M, 2015	Unilateral tibialis anterior	Ang-1	Up	Improve	[303]
Gattazzo F, 2014	Tibialis anterior	Cyclosporin A	Up	Improve	[304]
Kim MH, 2011	Unilaterally tibialis anterior	Akt	Up	Improve	[243]
Laziz I, 2007	Soleus	Spry	Down	——	[305]
Armand AS, 2003	Unilateral soleus	Follistatin and myostatin	Up/Down	Improve	[306]
Li C, 2013	Right gastrocnemius	Prosaposin	Up	——	[307]

**Table 7:** Non-SCs in the CTX-induced skeletal muscle injury models.

Author, Year	Injury Portions	Target Molecule/Drug	Regeneration	Ref
Kano K, 2020	Gastrocnemius	Capillary stem cells	Improve	[315]
Liu Y, 2007	Unilateral tibialis anterior	Flk-1+ AD-MSCs	Improve	[316]
Kim JA, 2013	Left tibialis anterior	hAFS cells transfected with MyoD	Improve	[318]
Xuan W, 2021	Tibialis anterior	Pluripotent stem cells-induced skeletal muscle progenitor cells with givinostat	Improve	[320]
Naldaiz-Gastesi N, 2019	Tibialis anterior	Human cremaster muscle-derived precursor cells	Improve	[321]
Mori J, 2008	Tibialis anterior	CD45+: Sca-1+ hematopoietic stem cells	Improve	[312]
Abedi M, 2007	Tibialis anterior	Hematopoietic stem cells	Improve	[322]
Hwang Y, 2014	Tibialis anterior	Human embryonic stem cells	Improve	[323]
Piccoli M, 2012	Tibialis anterior mice transplanted with bone marrow or amniotic fluid stem cells	Amniotic fluid stem cells	Improve	[324]
Yang R, 2010	Right tibialis anterior	Clones of ectopic stem cells	Improve	[325]
Rousseau J, 2010	EDL	Muscle precursor cells	Improve	[313]
Gang EJ, 2009	Tibialis anterior	Mesenchymal stem cells	Improve	[314]
Jung JE, 2017	Gastrocnemius and masseter	Pulp-derived cell	Improve	[310]
de la Garza-Rodea AS, 2011	Tibialis anterior	BM-hMSCs	improve	[311]
Bossolasco, 2004	Tibialis anterior	Human adult BM	Improve	[326]
Ma, 2012	Tibialis anterior	Human AF-amniotic fluid stem cells	Improve	[317]
Fukada S, 2002	Tibialis anterior	Bone marrow and fetal liver cells	Improve	[327]
Luth ES, 2008	Tibialis anterior, quadriceps, and gastrocnemius	Bone marrow side population cells	Improve	[328]
Zheng JK, 2006	Tibialis anterior	Human embryonic stem cells	Improve	[329]
Cížková D, 2011	Right tibialis anterior	BMCs	Improve	[308]
Meeson AP, 2004	Hindlimbs	Skeletal muscle side population	Improve	[330]
Drapeau C, 2010	Right tibialis anterior	Mobilization of bone marrow stem cells	Improve	[319]
Kowalski K, 2016	Gastrocnemius	Sdf-1 and granulocyte-colony stimulating factor	Improve	[331]
Mitchell R, 2019	Right tibialis anterior	ADSC secretome	Improve	[332]
Tobin S, 2021	Tibialis anterior; quadriceps; gastrocnemius	Young/aging macrophages	Improve/impair	[333]
